# Outstanding prognostic value of novel ferroptosis-related genes in chemoresistance osteosarcoma patients

**DOI:** 10.1038/s41598-022-09080-5

**Published:** 2022-03-23

**Authors:** Jiazheng Zhao, Yi Zhao, Xiaowei Ma, Helin Feng, Litao Jia

**Affiliations:** 1 Department of Orthopedics, The Fourth Hospital of Hebei Medical University, 12 Health Road, Shijiazhuang, Hebei 050011 People’s Republic of China; 2 Department of CT Scan, The Fourth Hospital of Hebei Medical University, 12 Health Road, Shijiazhuang, Hebei 050011 People’s Republic of China

**Keywords:** Cancer, Molecular biology

## Abstract

Osteosarcoma (OS) is the most common bone-derived tumor, and chemoresistance is a pivotal factor in the poor prognosis of patients with OS. Ferroptosis, as an emerging modality of regulated cell death, has demonstrated potential value in tumor chemoresistance studies. Through the gene expression omnibus database in conjunction with the FerrDb database, we identified novel ferroptosis-related differentially expressed genes (DEGs) involving chemoresistance in OS patients. Subsequently, enrichment analysis, protein–protein interaction network analysis and survival analysis were performed sequentially to recognize the hub genes and ultimately to construct a predictive model. The model constructed from the TARGET database was exhibited in a nomogram and assessed by calibration curves. The prognostic value of the model and hub genes was validated separately by an independent cohort. Twenty-two ferroptosis-related DEGs were identified, including 16 up-regulated and 6 down-regulated. Among them, expressions of CBS, COCS1, EGFR, as hub genes, were significantly associated with the prognosis of OS patients and were evidenced as independent prognostic factors. An efficient prognostic model covering hub gene expressions and clinical variables was developed and validated. Combining the results of hub genes in differential analysis, the actions of hub genes in ferroptosis, and the prognostic relevance of hub genes in patients, we revealed that CBS, SOCS1 and EGFR might play essential roles in OS and its chemoresistance with potential research and clinical value.

## Introduction

Osteosarcoma (OS), as the most prevalent tumor of bone origin, originates from primitive mesenchymal cells and mainly occurs in children and adolescents^1,2^. Chemotherapy, as the indispensable treatment option, has demonstrated a positive effect on the majority of patients with OS, but once chemoresistance strikes, it can lead to a dramatic reduction in patient survival^3,4^. The high susceptibility to chemotherapy resistance is also responsible for the lack of significant improvement in survival rates among OS patients over the past 20 years^5^. The specific mechanisms involved in the development of OS chemoresistance are not fully clarified, and protocols to address OS chemoresistance are urgently needed^6,7^.

Due to the fact that majority of tumor treatment regimens function by targeting apoptotic tumor cells, once tumor cells undergo apoptotic escape, treatment resistance will develop accordingly, leading to a disastrous prognosis^8,9^. Consequently, non-apoptotic forms of regulated cell death (RCD) associated with tumor therapy have come into the limelight and are gaining increased attention^10^. Distinct from apoptosis, ferroptosis, as a nontraditional RCD form featured by iron-dependent accumulation of lipid reactive oxygen species^11^, has been revealed to function as a pivotal role in tumor chemoresistance^12^. In particular, for OS, we identified through previous studies that ferroptosis may be tightly correlated with OS chemoresistance^13^. Ferroptosis is considered to be of great potential in antagonizing chemoresistance for OS^14^, however, further studies on the mechanisms associated with the action of ferroptosis on OS chemoresistance and the essential molecules involved are extremely rare.

In the present study, we obtained the list of ferroptosis-related genes and the characteristic of corresponding genes from the FerrDb database. In combination with the Gene Expression Omnibus (GEO) database, ferroptosis-related differentially expressed genes (DEGs) were acquired and followed by enrichment analysis and protein–protein interaction (PPI) network construction. Furthermore, hub genes with prognostic significance were identified through the TARGET database. The aim was to target critical ferroptosis-related DEGs by combining their own ferroptosis characteristics, expression characteristics and prognostic characteristics, and eventually construct an efficient prediction model whose prognostic value was individually validated by an independent cohort.

## Materials and methods

### Data sources and differential analysis

Through the GEO database (https://www.ncbi.nlm.nih.gov/geo/), we obtained RNA-seq data of 21 OS samples from the GSE87437 dataset based on GPL570 platform for differential analysis, including 11 chemoresistance samples and 10 non-chemoresistance ones. GEO2R (http://www.ncbi.nlm.nih.gov/geo/geo2r) was used to screen for DEGs meeting the criteria of *p* < 0.1, | log fold change (FC)|> 0.5. FerrDb (http://www.zhounan.org/ferrdb) is the first manually managed ferroptosis database covering regulatory factors and molecular markers for ferroptosis and ferroptosis-related diseases^15^. From the FerrDb database, 259 ferroptosis-related genes were downloaded, covering 108 drivers and 69 suppressors. In addition, 99 OS sample with survival information from the TARGET database (https://ocg.cancer.gov/ programs/target) were used for survival analysis and a prognostic model construction. For external validation of the prognostic value on the model and hub genes, through the GEO database, we obtained RNA-seq data and clinical information from the GSE21257 dataset based on GPL10295 platform, including 53 OS samples. All the above material was available from public databases and was free of ethical issue or informed consent.

### Enrichment analyses and PPI networks construction

Gene Set Enrichment Analysis (GSEA), Gene Ontology (GO) enrichment analysis, Kyoto Encyclopedia of Genes and Genomes (KEGG) enrichment analysis were conducted by the clusterProfiler package of R^16^. GO enrichment was applied to annotate and analyze genes involved in biological process (BP), cellular component (CC) and molecular function (MF)^17^. Moreover, we forecasted interactions between DEGs that achieved the combined score > 0.15 using Search Tool for the Retrieval of Interacting Genes/Proteins (STRING) database (https://string-db.org/)^18^ and visualized PPI networks using Cytoscape software, an open-source software for network analysis and visualization^19^. Top-ranked DEGs were calculated by cytoHubba^20^, a plug-in for Cytoscape to further screen hub genes.

### Survival analyses and model construction

The median of gene expression was used to divide the low and high expression groups, and overall survival was selected as the survival parameter. Survival analyses involving log-rank test, univariate Cox regression and multivariate Cox regression were performed using the survival package of R and visualized using the survminer package of R. Variables that were significant in the univariate analysis were included in the multivariate analysis and those that were significant in the multivariate analysis were further incorporated into the prognostic model. The rms package of R was used to develop a nomogram and calibration curves in order to construct and assess the prognostic model separately^21^. In addition, time-dependent receiver operating characteristic (ROC) curves were structured using the timeROC package of R to validate the prognostic value of the model and hub genes.

### Statistical analysis

Statistical analysis was conducted by version 3.6.3 of the R software and version 3.8.2 of the Cytoscape software. The analysis results were considered statistically significant at *p* < 0.05.

## Results

### Ferroptosis-related DEGs identification in chemoresistance OS patients

**Figure 1:**
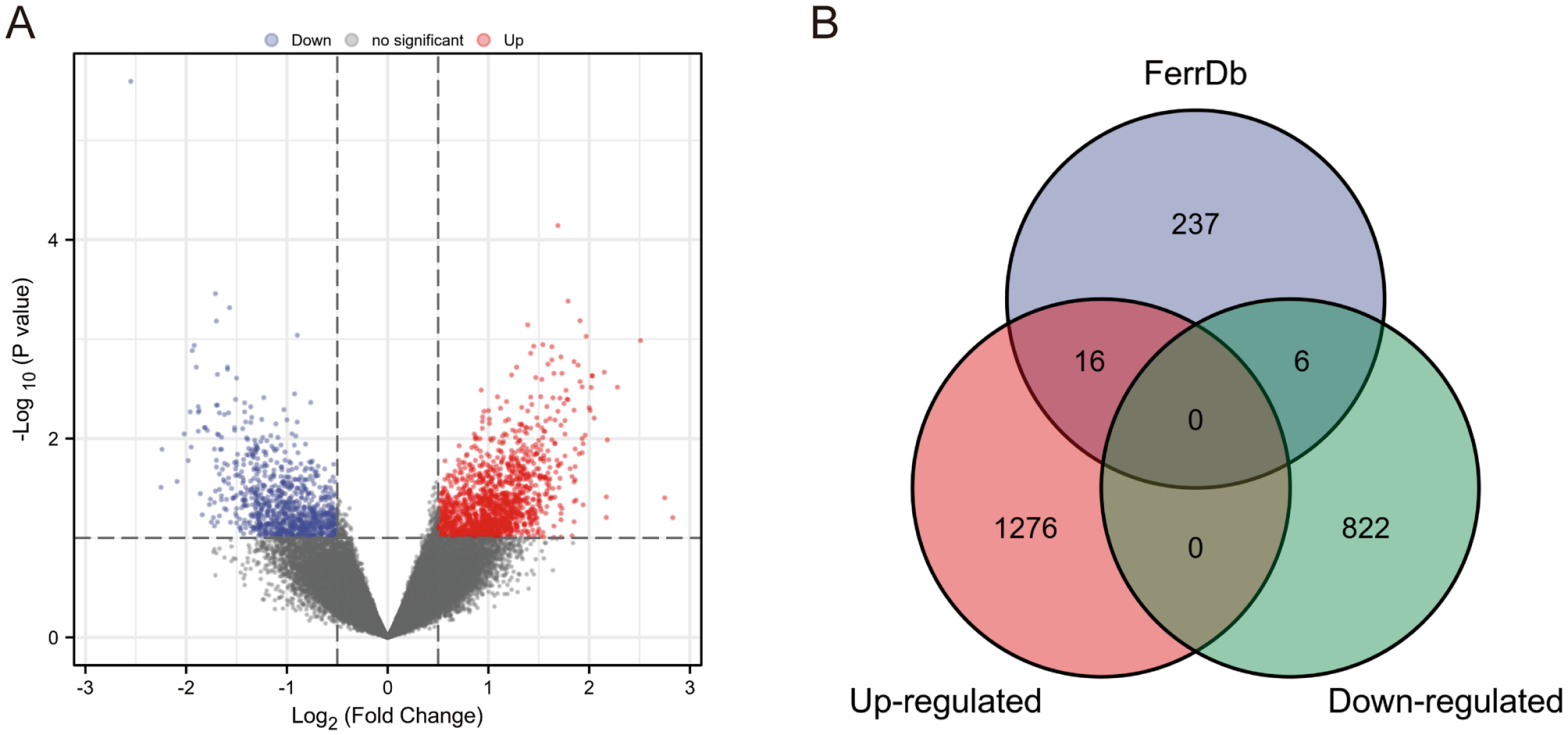
Ferroptosis-related DEGs identification in chemoresistance OS patients. (**A**) The volcano plot of differential analysis in the GSE87437 dataset. (**B**) The Venn diagram obtained by intersecting the DEGs in the GSE87437 dataset and ferroptosis-related genes in the FerrDb database.

Via the GEO database, we acquired RNA-seq data of 21 OS samples from the GSE87437 dataset for differential analysis, including 11 chemoresistance samples and 10 non-chemoresistance ones. The volcano plot presented the results of differential analysis via the GEO2R (Fig. 1A). Compared to non-chemoresistance samples, there were 1292 up-regulated DEGs and 828 down-regulated DEGs in chemoresistance ones. Among them, 22 ferroptosis-related DEGs were identified, including 16 up-regulated and 6 down-regulated ones (Table 1) (Fig. 1B).

**Table 1:** Ferroptosis-related DEGs in OS.

No	Genes	Expression	log FC
1	NOX1	Up-regulation	1.11
2	TFR2	Up-regulation	0.92
3	ALOX15B	Up-regulation	0.99
4	ACO1	Up-regulation	1.28
5	WIPI2	Up-regulation	0.75
6	EGFR	Up-regulation	1.21
7	SOCS1	Up-regulation	0.67
8	MUC1	Up-regulation	0.85
9	FANCD2	Up-regulation	0.91
10	PML	Up-regulation	1.21
11	TP63	Up-regulation	1.12
12	PROM2	Up-regulation	1.08
13	SLC2A6	Up-regulation	0.68
14	HNF4A	Up-regulation	1.24
15	TF	Up-regulation	1.23
16	ELAVL1	Up-regulation	0.82
17	ATG7	Down-regulation	−0.74
18	ALOX12B	Down-regulation	−1.01
19	SLC1A4	Down-regulation	−0.56
20	RGS4	Down-regulation	−2.09
21	SLC40A1	Down-regulation	−0.55
22	CBS	Down-regulation	−1.03

### GSEA of DEGs in chemoresistance OS patients

**Figure 2:**
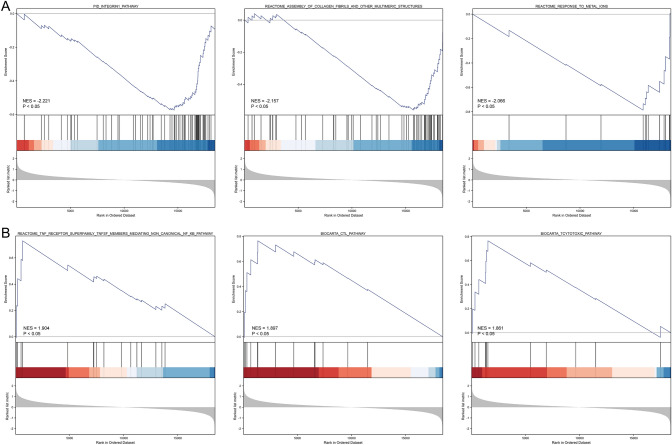
GSEA of ferroptosis-related DEGs in chemoresistance OS patients. (**A**) The top-three most significant-enriched gene sets that negatively correlated with the ferroptosis-related DEGs based on NES value. (**B**) The top-three most significant-enriched gene sets that positively correlated with the ferroptosis-related DEGs based on NES value.

The results of differential analysis in the GSE87437 dataset were subjected to GSEA (Table 2). The top-three most significant-enriched gene sets based on normalized enrichment score (NES) value, which negatively correlated with the DEGs, were integrin1 pathway, assembly of collagen fibrils and other multimeric structures, response to metal ions (Fig. 2A). Besides, the top-three most significant-enriched gene sets based on NES value, which positively correlated with the DEGs, were TNFRSF members mediating non canonical NF-kappaB pathway, CTL pathway, tcytotoxic pathway (Fig. 2B).

**Table 2:** Top-ten most significant-enriched gene sets according to NES value ranking of GSEA results positively and negatively correlated with DEGs, respectively.

No	Significant-enriched genes sets	Correlation	NES
1	REACTOME_TNF_RECEPTOR_SUPERFAMILY_TNFSF_MEMBERS_MEDIATING_NON_CANONICAL_NF_KB_PATHWAY	Positive	1.90
2	BIOCARTA_CTL_PATHWAY	Positive	1.90
3	BIOCARTA_TCYTOTOXIC_PATHWAY	Positive	1.86
4	REACTOME_UNBLOCKING_OF_NMDA_RECEPTORS_GLUTAMATE_BINDING_AND_ACTIVATION	Positive	1.85
5	REACTOME_ROLE_OF_PHOSPHOLIPIDS_IN_PHAGOCYTOSIS	Positive	1.84
6	BIOCARTA_NO2IL12_PATHWAY	Positive	1.82
7	REACTOME_PIWI_INTERACTING_RNA_PIRNA_BIOGENESIS	Positive	1.80
8	BIOCARTA_THELPER_PATHWAY	Positive	1.79
9	REACTOME_SURFACTANT_METABOLISM	Positive	1.78
10	REACTOME_OLFACTORY_SIGNALING_PATHWAY	Positive	1.77
11	PID_INTEGRIN1_PATHWAY	Negative	−2.22
12	REACTOME_ASSEMBLY_OF_COLLAGEN_FIBRILS_AND_OTHER_MULTIMERIC_STRUCTURES	Negative	−2.16
13	REACTOME_RESPONSE_TO_METAL_IONS	Negative	−2.07
14	REACTOME_COLLAGEN_FORMATION	Negative	−2.06
15	WP_CANONICAL_AND_NONCANONICAL_TGFB_SIGNALING	Negative	−2.02
16	REACTOME_SIGNALING_BY_NODAL	Negative	−2.02
17	WP_MIRNA_TARGETS_IN_ECM_AND_MEMBRANE_RECEPTORS	Negative	−2.00
18	REACTOME_COLLAGEN_BIOSYNTHESIS_AND_MODIFYING_ENZYMES	Negative	−1.98
19	REACTOME_CROSSLINKING_OF_COLLAGEN_FIBRILS	Negative	−1.97
20	REACTOME_LAMININ_INTERACTIONS	Negative	−1.94

### GO and KEGG enrichment analyses of ferroptosis-related DEGs

**Figure 3:**
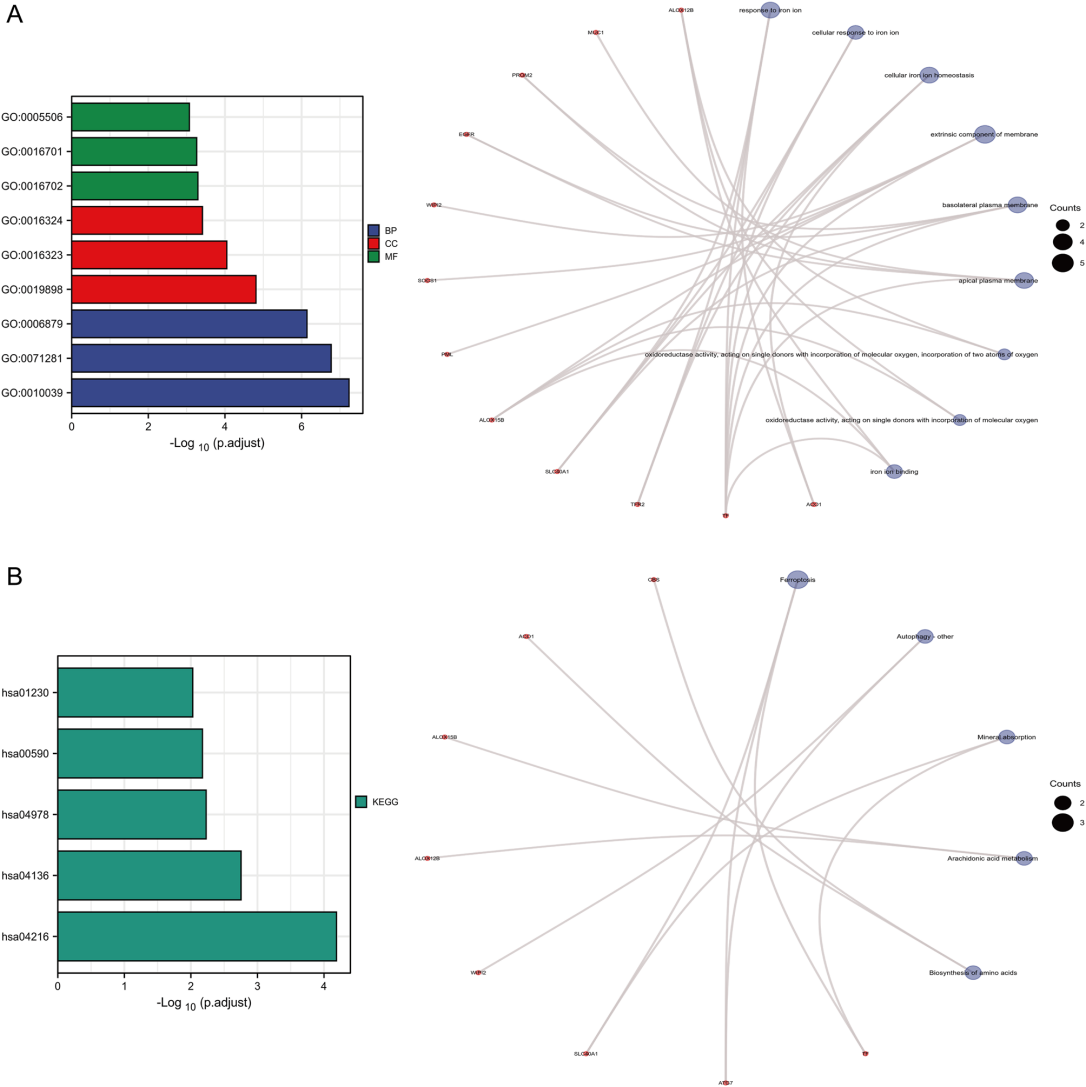
GO and KEGG enrichment analyses of ferroptosis-related DEGs. (**A**) GO enrichment analysis visualization network based on the top-three most significant-enriched terms in each of the BP, CC, and MF entries. (**B**) KEGG enrichment analysis visualization network based on the top-five most significant-enriched pathways.

GO and KEGG enrichment analyses were performed on 22 ferroptosis-related DEGs. GO analysis suggested that these genes mainly functioned in response to iron ion, extrinsic component of membrane, and oxidoreductase activity. The top-three most significant-enriched terms in each of the BP, CC, and MF entries were identified for GO visualization network construction (Fig. 3A). In addition, KEGG analysis indicated that corresponding genes were associated with ferroptosis, autophagy, mineral absorption, arachidonic acid metabolism, and biosynthesis of amino acids. The top-five most significant-enriched pathways were identified for KEGG visualization network construction (Fig. 3B).

### PPI networks of ferroptosis-related DEGs

**Figure 4:**
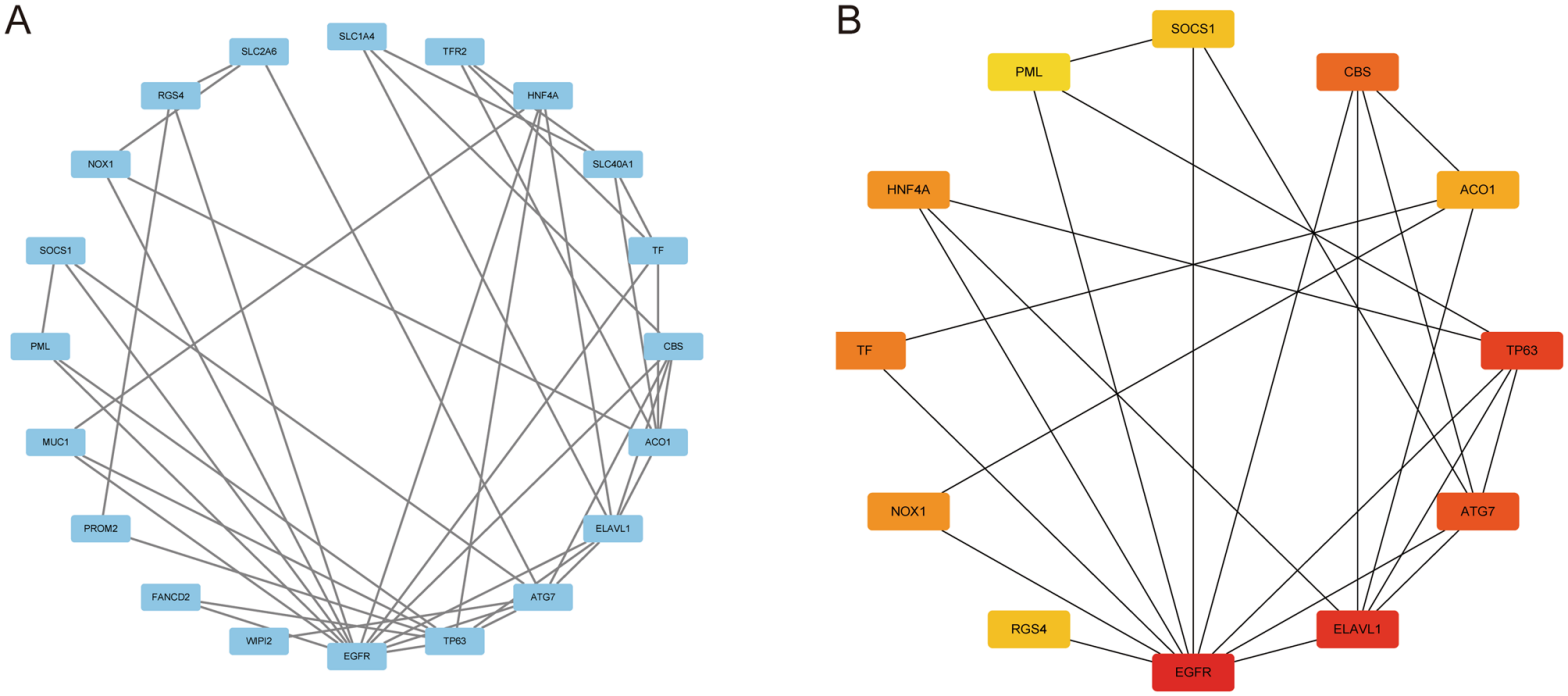
PPI networks of ferroptosis-related DEGs. (**A**) The initial PPI network covering 20 nodes and 43 edges using Cytoscape. (**B**) The PPI network covering 12 nodes and 24 edges after filtering by cytoHubba.

Interactions between ferroptosis-related DEGs were predicted using STRING database with a combined score > 0.15 and visualized using Cytoscape software. The initial PPI network with 20 nodes and 43 edges was developed (Fig. 4A). Furthermore, we applied cytoHubba to identify the top-ranked genes and recognized a 12-node, 24-edge PPI network based on the initial network (Fig. 4B).

### Hub genes identification

**Figure 5:**
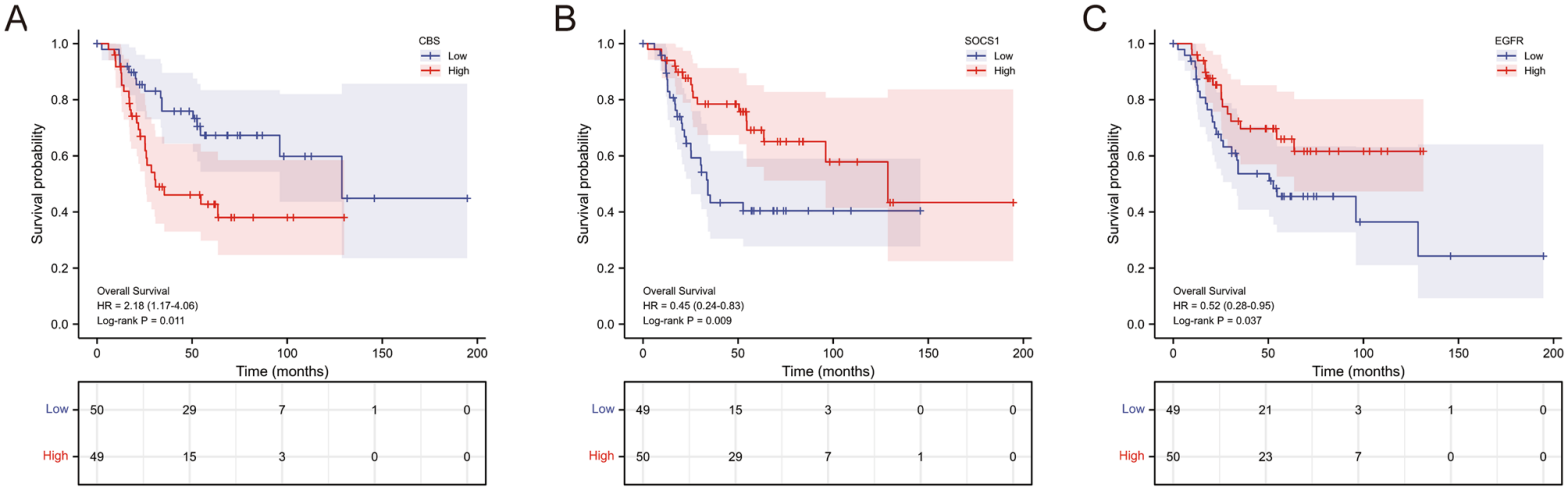
K-M curves of hub genes for OS patients in the TARGET database. (**A**) High expression of CBS was significantly associated with poor survival. (**B**) Low expression of SOCS1 was significantly associated with poor survival. (**C**) Low expression of EGFR was significantly associated with poor survival.

Among these genes, we discovered that the expression of cystathionine beta-synthase (CBS), suppressor of cytokine signaling 1 (SOCS1) and epidermal growth factor receptor (EGFR) influenced the prognosis of OS patients in TARGET database. High expression of CBS (Fig. 5A), and low expression of SOCS1 (Fig. 5B), EGFR (Fig. 5C) were significantly associated with poor survival probability. Meanwhile, CBS, a suppressor of ferroptosis, was up-regulated in chemoresistance samples according to previous differential analysis, while SOCS1 and EGFR, as drivers of ferroptosis, were correspondingly down-regulated in chemoresistance samples. We targeted these three genes as hub genes for further analyses.

### Expressions of CBS, SOCS1 and EGFR might be independent prognostic factors for OS patients

Through the TARGET database, clinical characteristics of OS patients (containing age, gender, race, surgery, metastasis, tumor side, tumor region, primary site progression) together with hub gene expressions were included in univariate Cox regression analysis. The results showed that metastasis, high expression of CBS, low expression of SOCS1 and EGFR were risk factors for poor prognosis in patients with OS. Furthermore, these variables with significant results were integrated into multivariate Cox regression analysis, which revealed that expressions of CBS, SOCS1 and EGFR might be independent prognostic factors for OS patients (Table 3).

**Table 3:** Cox regression analyses of overall survival for OS patients.

Variables	Total (N)	Univariate analysis		Multivariate analysis	
		Hazard ratio (95% CI)	*p* value	Hazard ratio (95% CI)	*p* value
Age (< 18 vs. > = 18)	99	1.365 (0.605–3.081)	0.454		
Gender (female vs. male)	99	1.024 (0.546–1.922)	0.940		
Race (white vs. others)	75	0.754 (0.315–1.803)	0.525		
Surgery (limb sparing vs. amputation)	58	1.376 (0.410–4.613)	0.606		
Tumor side (right vs. left)	27	1.321 (0.494–3.529)	0.579		
Primary site progression (yes vs. no)	50	1.769 (0.864–3.626)	0.119		
Tumor region (distal vs. proximal)	60	2.321 (0.925–5.823)	0.073		
Metastasis (yes vs. no)	99	3.679 (1.964–6.892)	< 0.001	3.564 (1.883–6.743)	< 0.001
EGFR (low vs. high)	99	1.942 (1.028–3.669)	0.041	2.203 (1.122–4.327)	0.022
SOCS1 (low vs. high)	99	2.274 (1.207–4.283)	0.011	2.323 (1.202–4.488)	0.012
CBS (high vs. low)	99	2.217 (1.176–4.180)	0.014	2.601 (1.321–5.118)	0.006

### Validation for the prognostic value of the hub genes

**Figure 6:**
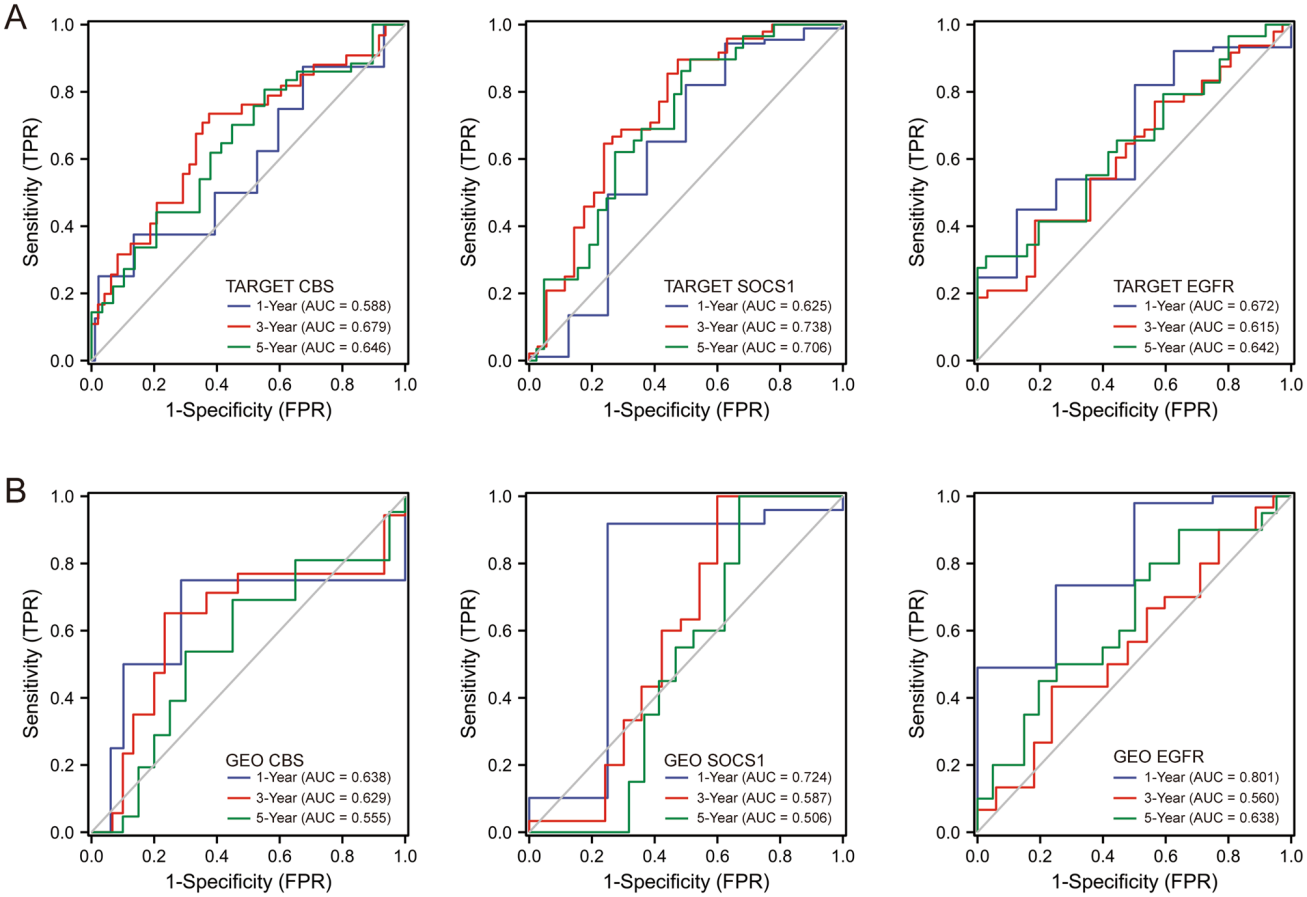
Time-dependent ROC curves of hub genes for OS patients. (**A**) Internal validation via a cohort of 99 samples from the TARGET database. (**B**) External validation via a cohort of 53 samples from the GSE21257 dataset.

Internal validation was performed through the TARGET database to detect the prognostic value of the 3 hub genes with time-dependent ROC curves (Fig. 6A). Moreover, external validation for the prognostic value of CBS, SOCS1 and EGFR was implemented in an independent cohort of 53 OS samples from the GSE21257 dataset, which demonstrated the results consistent with the internal validation (Fig. 6B).

### A prognostic model construction and evaluation

**Figure 7:**
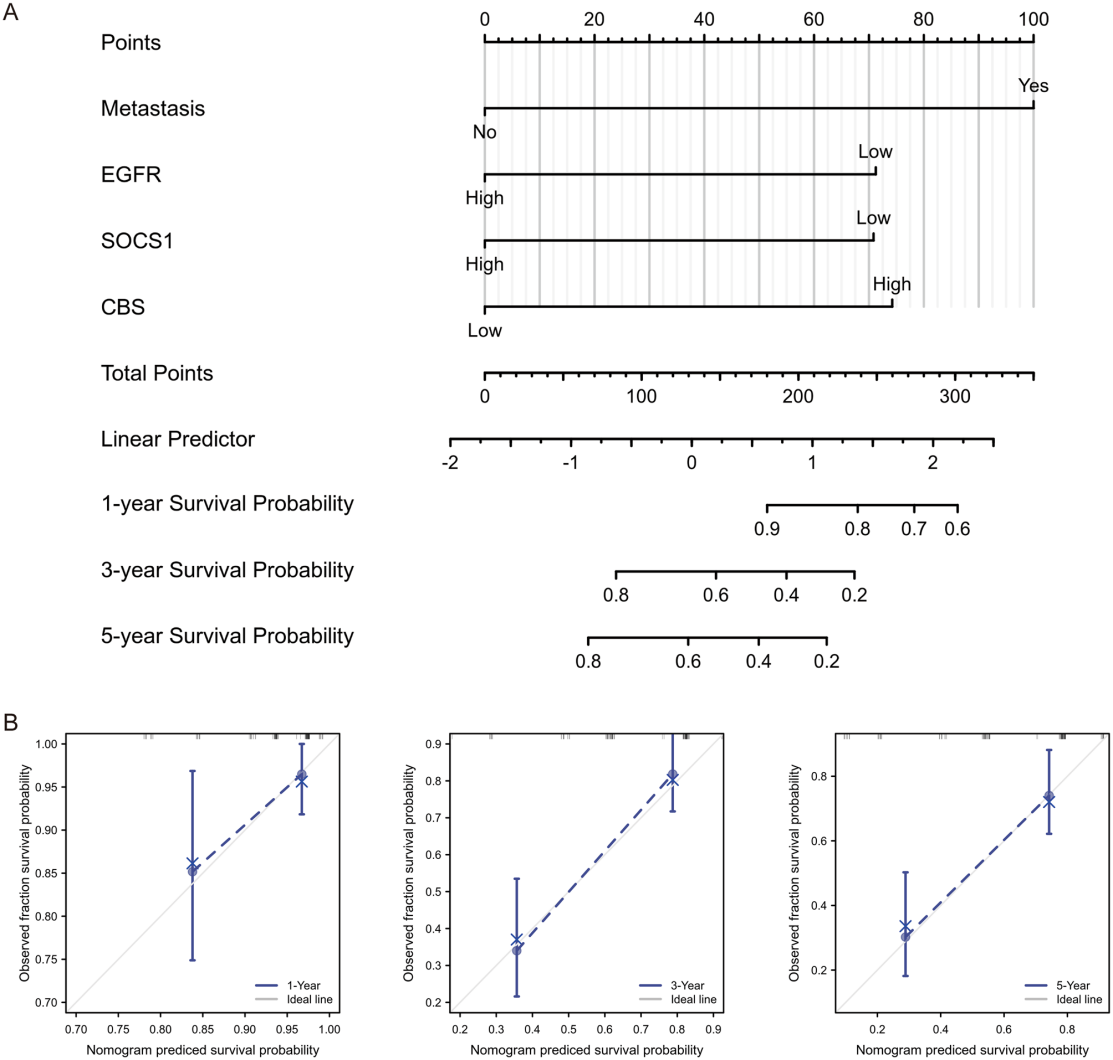
Visualization of the prognostic model for OS patients. (**A**) Construction of a nomogram to predict 1-, 3-, and 5-year survival probability. (**B**) Construction of 1-, 3-, and 5-year calibration curves to evaluate predictive efficacy of the nomogram.

Variables with significant results in multivariate Cox regression analysis were incorporated into the construction of the prognostic model. Metastasis and expressions of CBS, SOCS1, EGFR were aggregated in a nomogram to predict 1-, 3-, and 5-year survival probability in patients with OS (Fig. 7A). The C-index of this prognostic model reached 0.788 (0.756–0.820), and the predicted and actual outcomes in the 1-, 3-, and 5-year calibration curves were nearly identical (Fig. 7B).

### Validation for the prognostic value of the model

**Figure 8:**
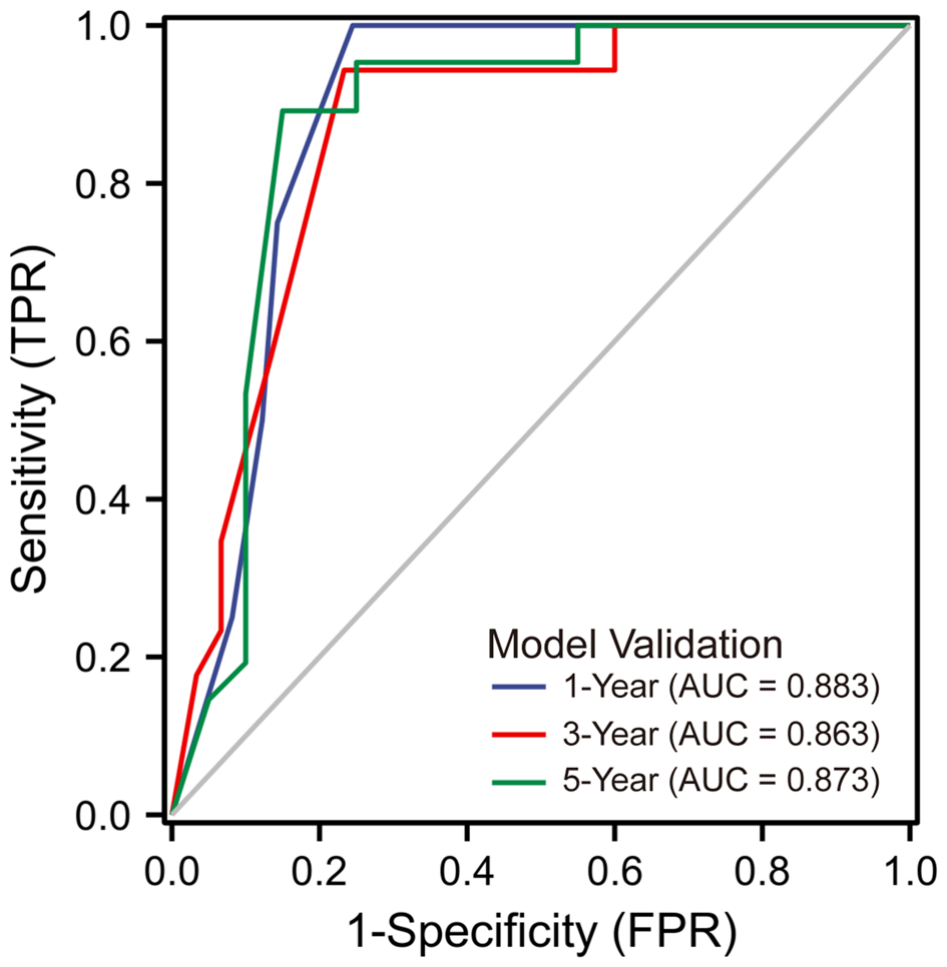
Based on an independent external validation cohort of 53 OS samples from the GSE21257 dataset, the time-dependent ROC curve demonstrated the model's outstanding predictive efficacy for prognosis of patients with OS.

An independent cohort of 53 OS samples from the GSE21257 dataset was conducted to externally validate the model. The time-dependent ROC curve demonstrated the model's outstanding predictive efficacy for prognosis of patients with OS, and was higher than that of individual hub genes (Fig. 8).

## Discussion

We have previously reported that ferroptosis can impact chemotherapy resistance in patients with OS^13^. Chemotherapy, a common management for cancer, provides varying degrees of therapeutic efficacy for most human cancers^22^. However, since the majority of the treatment regimens involved target the apoptotic effect of tumor cells, once apoptotic escape of tumor cells occurs, it can lead to a reduction in the sensitivity of the original treatment and thereby resulting in a poor prognosis^23^. Distinguished from apoptosis, ferroptosis, as an emerging type of RCD with unique morphological and biological characteristics, is recognized as promising in modifying cancer chemoresistance and has demonstrated corresponding value in OS chemotherapy^24^. Fu et al. observed that OS cells that underwent ferroptosis behaved more sensitively to adriamycin treatment^25^. Liu et al. revealed a significant decrease of ferroptosis activity in cisplatin-resistant OS cells, while non-resistant ones gradually developed a hyposensitivity to cisplatin along with the addition of ferroptosis inhibitors, and this hyposensitivity could be ameliorated by ferroptosis agonists^12^. In the present study, we identified 22 ferroptosis-related DEGs between chemoresistance and non-chemoresistance samples of OS, from which CBS, COCS1, EGFR were detected to be associated with overall survival in OS patients, and thus they were classified as hub genes for further study to explore the potential molecular mechanism and prognostic value. The aim was to target critical ferroptosis-related DEGs by combining their own ferroptosis characteristics, expression characteristics and prognostic characteristics, and eventually construct an efficient prediction model whose prognostic value was individually validated by an independent cohort.

Through the TARGET database, high expression of CBS and low expression of SOCS1, EGFR suggested poor survival probability for patients with OS. CBS, shown to be up-regulated in chemoresistant samples by differential analysis, is regarded as a suppressor of ferroptosis^26,27^. CBS is the rate-limiting enzyme as well as the first enzyme in the transsulfuration pathway, which serves a critical role for humans in the maintenance of health and the development of disease^28^. The typical biochemical function of CBS under physiological conditions is to catalyze the transition of serine and homocysteine to cystathionine and water. Additionally, CBS can also catalyze substitution reactions which generate hydrogen sulfide^29,30^. Suppression of CBS has been reported to initiate ferroptosis in hepatocellular carcinoma^27^. A similar response was measured by Liu et al. in ovarian cancer cells, whereby knockdown of CBS enhanced ferroptosis susceptibility of cancer cells, while overexpression of CBS correspondingly promoted ferroptosis resistance^26^. In addition, SOCS1 and EGFR, shown to be down-regulated in chemoresistant samples by differential analysis, are considered as drivers of ferroptosis^31,32^. SOCS1 belongs to a family of 8 intracellular proteins that under physiological conditions limit type I Interferon and Interferon-γ receptor activation, serving as a classical negative feedback loop regulator of the Janus kinase and – signal transducer and activator of transcription pathway^33,34^. SOCS1 has exhibited significant potential in multiple diseases, and its related gene therapy and biologic application are active fields of study worldwide^35^. For a wide range of cancers, SOCS1 is recognized as a tumor suppressor and may act in a cell context-dependent manner^36^. As for OS, it has been confirmed that expression of SOCS1 can sensitize U-2 OS cells to ferroptosis inducer, which may be linked to the reduced glutathione level^32^. EGFR is a transmembrane glycoprotein belonging to the tyrosine kinase receptor family, whose binding to cognate ligands result in its autophosphorylation and subsequent activation of signal transduction pathways involved in regulating cell proliferation, differentiation and survival^37,38^. In terms of EGFR in ferroptosis, Poursaitidis et al. discovered that cell death in activated EGFR mutant cells occurred by ferroptosis and repression of EGFR signaling pathway corresponded to the rescue of cell viability^31^. Apart from its broad role in the genesis and progression of tumors, EGFR also influences therapeutic efficacy and is engaged in chemoresistance, which has recently been described as involving ferroptosis effect^39^. Combining the results of hub genes in differential analysis, the actions of hub genes in ferroptosis, and the prognostic relevance of hub genes in patients, we considered that CBS, SOCS1 and EGFR might play essential roles in OS and its chemoresistance with potential research and clinical value.

Furthermore, we included hub gene expressions along with clinical variables for patients with OS in Cox regression analyses and identified variables with statistically significant results, containing expressions of CBS, SOCS1, EGFR and metastasis, for incorporation into the construction of a prognostic model. OS tends to indicate a high malignancy degree and a poor survival period, and given its complexity and heterogeneity, accurate prediction of patient prognosis is in high demand^40^. More clinical models of OS have been reported in successive studies. Cao et al. developed the prognostic model for OS on the basis of immune-related genes^41^. Zhang et al. used genes identified by co-expression network analysis as the predictive tool for OS lung metastasis^42^. Xiao et al. assessed the prognosis of OS patients in terms of key macrophage-associated genes^43^. In the present study, the value of both the model and the adopted genes were validated in an independent cohort, and the near overlap between predicted and actual outcomes in the 1-, 3-, 5-year calibration curves suggested high efficiency. This is one of the few studies on OS chemoresistance involving ferroptosis and the first to construct the prognostic model for OS patients with ferroptosis-related genes. However, there remain certain limitations to our study. On the one hand, the low incidence of OS and the restrictive condition of chemoresistance have led to a relatively small number of samples for differential analysis, which may result in the omission of some valuable genes. On the other, numerous clinical studies as well as cellular and animal experiments are required to support our findings.

In conclusion, we revealed CBS, COCS1, and EGFR as ferroptosis-related genes that might be critically involved in OS chemoresistance and applied them to construct an efficient predictive model for the prognosis of OS patients.

## Data Availability

All data used, including gene expression, sequences and patient clinical information, are available from the GEO database (https://www.ncbi.nlm.nih.gov/geo/) (GSE87437, GSE21257) and the TARGET database (https://ocg.cancer.gov/programs /target). All data were obtained from public databases and was free of ethical issue or informed consent.
